# An Infrastructure to Mine Molecular Descriptors for Ligand Selection on Virtual Screening

**DOI:** 10.1155/2014/325959

**Published:** 2014-04-09

**Authors:** Vinicius Rosa Seus, Giovanni Xavier Perazzo, Ana T. Winck, Adriano V. Werhli, Karina S. Machado

**Affiliations:** ^1^Centro de Ciências Computacionais, Universidade Federal do Rio Grande - FURG, Avenida Itália km 8 s/n, 96203-900 Rio Grande, RS, Brazil; ^2^Departamento de Computação Aplicada, Universidade Federal de Santa Maria - USFM, Avenida Roraima 1000, 97105-900 Santa Maria, RS, Brazil

## Abstract

The receptor-ligand interaction evaluation is one important step in rational drug design. The databases that provide the structures of the ligands are growing on a daily basis. This makes it impossible to test all the ligands for a target receptor. Hence, a ligand selection before testing the ligands is needed. One possible approach is to evaluate a set of molecular descriptors. With the aim of describing the characteristics of promising compounds for a specific receptor we introduce a data warehouse-based infrastructure to mine molecular descriptors for virtual screening (VS). We performed experiments that consider as target the receptor HIV-1 protease and different compounds for this protein. A set of 9 molecular descriptors are taken as the predictive attributes and the free energy of binding is taken as a target attribute. By applying the J48 algorithm over the data we obtain decision tree models that achieved up to 84% of accuracy. The models indicate which molecular descriptors and their respective values are relevant to influence good FEB results. Using their rules we performed ligand selection on ZINC database. Our results show important reduction in ligands selection to be applied in VS experiments; for instance, the best selection model picked only 0.21% of the total amount of drug-like ligands.

## 1. Introduction

One of the most important steps in rational drug design (RDD) is the receptor-ligand interaction evaluation at an atomic level, which is achieved through molecular docking simulations [1]. This is an* in silico* step that accelerates the new drug discovery process. In these simulations a docking algorithm predicts the best position and conformation of a drug candidate (small molecule, compound or ligand) within the constraints of a target receptor binding site in order to correctly estimate their stability in terms of free energy of binding scores [1, 2].

In the early stages of the drug discovery process researchers can be interested not only in understanding the interaction between one receptor-ligand but also in testing a set of different drug candidates in a process defined as structured based virtual screening (VS) [3]. This VS technique for identifying hit molecules is an important starting point in the search for new inhibitors [3]. The ligands or compounds can be obtained from different databases as ZINC [4] and PubChem [5]. These repositories are growing daily at a high rate, providing continuously more structures for improving the quality of the VS experiments. However, this growth makes it impossible to test all the available compounds into a target receptor. Hence, it is essential to select the most promising compounds before testing them* in silico*. This selection can be performed with different approaches. For instance, it may make use of molecular coordinates or consider a set of molecular properties, also called molecular descriptors.

With the aim to contribute to a more effective ligand selection, in this work we focus on a new strategy to easily and efficiently describe important characteristics that indicate promising compounds to be investigated in VS experiments. Initially we proposed data warehouse (DW) schemas which are able to integrate molecular descriptors from different databases and relate them with VS experimental data [6]. With this powerful infrastructure we are encouraged to propose a methodology that makes use of the stored data to perform mining experiments on molecular descriptors characteristics. Such a methodology, which is based on decision trees algorithm, aims at pointing out features related to molecular descriptors that in turn will lead to good free energy of binding (FEB) values on molecular docking experiments.

To validate our proposed infrastructure, we performed a VS case study using AutoDock4 [7] which considers as target the receptor HIV-1 protease and 76 previously known promising compounds for this protein (experiment 1). For effectively extracting rules from the decision trees to select ligands we performed another VS case study (experiment 2) considering 410 compounds and the same target protein. Having these data stored in our data warehouse we could generate the appropriate input data mining files. These data sets obtained from our DW are composed by 9 molecular descriptors and the free energy of binding (FEB). The FEB is obtained from molecular docking experiments and is used as target attribute.

Our approach to preprocess the input files is primordial to achieve the expected data mining results. Over these input files we applied the J48 decision tree algorithm achieving up to 75% of accuracy in experiment 1 and up to 84% in experiment 2. From the induced models of experiment 2 we extracted rules used to effectively select ligands from ZINC database. The rule that produced the smallest selection picked only 0.21% of the total amount of drug-like ligands in ZINC, while the rule that resulted in the largest selection picked 25% of this total. In face of this, our results show important reduction in ligand selection to be applied in VS experiments. Despite the stored data, our DW is sufficiently generic to store as many structures, molecular descriptors, and molecular docking experiments as needed. Likewise, our data mining methodology is generic enough to be applied over any data stored in the proposed DW.

The remaining of this work is structured as follows.  Section 2 describes the material and methods including the description of virtual screening and molecular docking detailing the target receptor and ligands used on the case study. Also on Section 2 we present the proposed infrastructure. In this infrastructure subsection we detailed the considered molecular descriptors and the developed data warehouse.  Section 3 presents the results and discussion including the two performed VS experiments and the ligand selection step. Finally on Section 4 we conclude the paper and describe the future work.

## 2. Materials and Methods

### 2.1. Classification Task and Decision Trees

According to Tan et al. [8] classification is a data mining task of assigning objects to one of several predefined categories. The input data for classification is a training set composed by a collection of records characterized by a tuple (*x*, *y*). In this tuple *x* is a set of predictive attributes and *y* is the class label (also known as target attribute or category). The learning step in classification task builds a model *f* where each attribute in *x* is mapped to one of the predefined discrete-valued and unordered target attribute *y* [8, 9]. There are many different classification algorithms, for instance, support vector machines, neural networks, naive Bayes, and the decision trees.

Decision trees output is a flowchart-like tree structure in which the internal nodes denote a test on an attribute, each branch represents an outcome of the test and each leaf node is assigned a class label [9]. According to Freitas et al. [10] this output graphically represents the discovered knowledge being easily understandable by researchers from different areas. Besides this kind of classification model points out to the importance of the attributes used for prediction [10].

Decision trees can be used for classification since, given a tuple *x* for which the class label is unknown, the attribute values of *x* are tested against the decision tree and the path traced defined the prediction class [11]. In doing so, we decided to apply the C4.5 [12] classification decision tree algorithm (WEKA J48 implementation [11]).

### 2.2. Virtual Screening and Molecular Docking

Rational drug design [13] has been applied in order to accelerate the drug discovery process. It is an important step because the costs and time involved in the discovery of a new drug for a specific target are constantly increasing [14]. The RDD methodology can be based on the three-dimensional target receptor structure. In this case, the starting point is to know the target receptor structure and consequently its binding site. Based on the binding or active site an inhibitor candidate (or ligand) can be bounded to a stable complex.

Virtual screening (VS) is an* in silico* technique where a set of large libraries of drug candidates are analyzed in order to identify those structures which are most likely to bind to a receptor target, typically a protein or an enzyme [15]. The structure-based virtual screening involves molecular docking simulation of candidate ligands into a receptor target applying a scoring function to estimate with which affinity the ligand will bind to the receptor. This affinity is measured by the free energy of binding (FEB), where the lower values correspond to better receptor-ligand complexes. Then, the best candidates are experimentally tested and the next steps of a RDD process are performed.

To perform the experiments of this work we had to choose some tools and databases. There are many molecular docking softwares, for example, FlexE [16], Gold [17], and AutoDock [7]. AutoDock is a popular and efficient docking tool that we have used previously in docking experiments. For these reasons we choose to consider this tool in our work.

Besides the molecular docking tool, the VS strategy involves the use of ligand databases. As reviewed in [18] the most important public compounds database is ChemBank [19], ChemDB [20], NCI Database [21], PubChem [22], and ZINC [4]. In this work we choose to use the ZINC database since it is a free database of commercially available compounds that contains over 22 million purchasable compounds ready for molecular docking.

#### 2.2.1. The Protein and the Ligands

**Figure 1:**
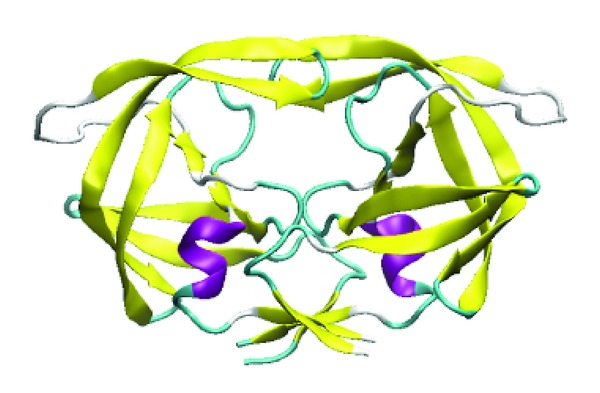
The three-dimensional structure of the HIV-1 protease target receptor (PDB Code: 1HPV).

In order to validate our proposed methodology we performed a case study with two experiments considering as the target receptor the protein HIV-1 protease (PDB Code: 1HPV) [23], which is illustrated in Figure 1. HIV-1 protease (HIV PR) is a retroviral protease that is essential for the life cycle of HIV, the retrovirus that causes immunodeficiency syndrome (AIDS) [23, 24]. The inhibition of the HIV-1 protease activity disrupts HIV-1 ability to replicate and infect additional cells making this protein inhibition the subject of innumerous pharmaceutical research [24].

In the first case study (experiment 1) we have used 76 out of 100 ligands considered in [25] obtained from ZINC. We decided not to use all the 100 ligands because for some of them we have not found the corresponding ZINC entry on ZINC database. Thus, to perform our VS experiments we used AutoDock4 and the available scripts as described in Lindstrom et al. [25]. Based on the characteristics of the best molecular docking results of the first experiment we selected a new set of 410 ligands also obtained from ZINC database.

### 2.3. Infrastructure

**Figure 2:**
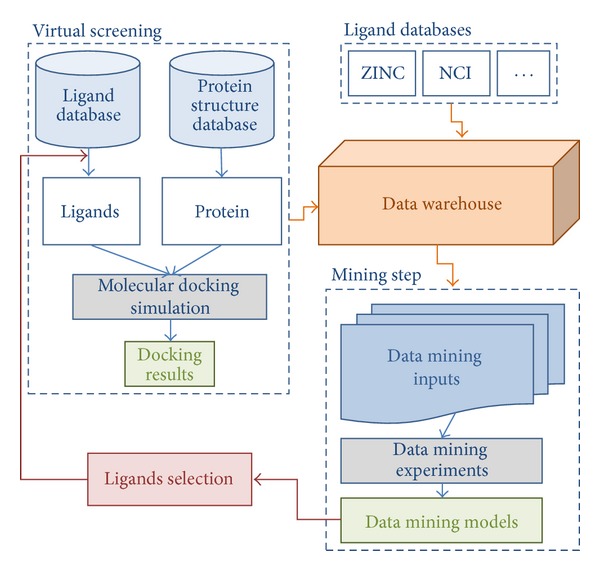
Infrastructure to mine molecular descriptors for virtual screening. The structure is composed by 5 major interactive modules: virtual screening, ligand databases, data warehouse, mining, and ligand selection.

The proposed infrastructure applied for mining molecular descriptors for virtual screening is depicted in Figure 2. It is composed by 5 major interactive modules: virtual screening, ligand databases, data warehouse, mining, and ligand selection.

In the virtual screening module we collect both data from ligands in public ligand databases and from proteins structures in a PDB format. In these ligands and protein we perform molecular docking simulations and hold the respective results. From the diverse ligand databases we collect the molecular descriptors for each ligand being used. All data related to virtual screening and ligand databases modules are properly processed and inserted into the DW we have developed [6]. The stored data can then be preprocessed so that we can start the mining experiments. To achieve this we produce suitable input files for data mining experiments creating models that indicate whether and how a given molecular descriptor can have influence on good FEB values in docking experiments. Based on the characteristics identified on the data mining models we can perform ligand selection on the ligand databases for new virtual screening experiments.

#### 2.3.1. Molecular Descriptors

In databases repositories of ligands we can find different information about compounds and such information can be very relevant for virtual screening. A wide number of this type of repositories are available for different research purposes. Among them we can mention the Cambridge Structural Databases (CSD) [26], ChemBank [19], ChemDB [20], MMsINC Database [27], NCI Database [21], PubChem [22], and ZINC [4]. Apart from CSD repository which is private all the other repositories listed before are public and do not hold a license charge.

All these databases store a different set of ligands. All of them provide for the stored ligands both their spatial coordinates and a set of properties or molecular descriptors. These properties are encoded information from the molecular structures where the molecular descriptors become numerical values representing such information [28, 29].

In [18] it is possible to find a comparison among the public ligand databases. A set of 10 features were evaluated for each of the public ligand databases mentioned before, with the aim identifying which of them is the most comprehensive and suitable for VS. The authors pointed out ZINC as the most suitable public ligand database in terms of features availability. ZINC is a database made available since 2005 and currently stores over than 21 million molecules, with a set of 9 molecular descriptors to describe them. Even though other public molecular databases store molecular descriptors in their own way, here we depict the 9 molecular descriptors presented in ZINC and that are considered in experiment 1. Besides, we can mention that the first 5 molecular descriptors listed below match with the ones in the 6 public ligand databases cited before:

- molecular weight (MwT);
- predicted octanol-water partition coefficient (log⁡*P*);
- number of hydrogen bond donors (HBD);
- number of hydrogen bond acceptors (HBA);
- number of rotatable bonds (NRB);
- apolar desolvation energy (ADE);
- polar desolvation energy (PDE);
- total polar surface area (TPSA);
- charge (Ch).

#### 2.3.2. Data Warehouse

Data warehouse can be defined as a repository holding information from multiple sources [9]. This information is stored under a unified schema to facilitate decision making and built in a way to satisfy a multidimensional structure [30], also called analytical model. We have introduced a DW schema able to integrate molecular descriptors from different public ligand databases as well as able to relate them with virtual screening experiments data [6]. The main idea of our DW is not only to provide a single source capable of storing as many molecular descriptors as the ones provided by different public ligand databases but also to provide historic records of virtual screening experiments using molecular descriptors.

The mentioned DW [6] contains 6 dimension tables to represent the subject we are modelling, around a central fact table, called virtual screening. This DW schema can provide data about characteristics appearing as relevant in a virtual screening experiment, shared in the dimension tables about the whole experiment; molecular descriptors; database from with structures were collected; proteins; ligands and atoms. All the dimension tables are structured around the major term, the fact table, which here is represented by the virtual screening result itself. That is, it holds all ID from the dimensions, plus values that determine the quality of the docking experiments: FEB and root mean square deviation (RMSD) values. The different kinds of data format one can retrieve from the DW allow us to build proper data sets for mining experiments.

#### 2.3.3. Data Sets

In order to validate our proposed methodology and DW we performed a case study considering the HIV-1 protease receptor and 76 ligands as we described on Section 2.2.1. This case study is experiment 1. For experiment 2 we performed the case study considering 410 ligands described on Section 2.2.1. After preparing the receptor and ligands entries we performed VS experiment using AD4 software where we choose for both experiments the Lamarckian genetic algorithm with the following parameters: 10 runs, 10 individuals in population, maximum number of energy evaluations defined as 250,000, and maximum number of generations set to 27,000.

Following the previous steps the results were stored on DW tables:

- Protein stores only the information about the HIV-1 protease receptor;
- Mol descriptor, database, ligand, and atom save the information about the 76 used ligands (considered in experiment 1), comprising their structures, molecular descriptors, and provenance data;
- Mol descriptor, database, ligand, and atom save the information about the 410 used ligands (considered in experiment 2), comprising their structures, molecular descriptors, and provenance data;
- Experiments record the molecular docking simulation results.

Thus, using the stored data in our DW we generated the appropriate data mining inputs. In our case study of experiments 1 and 2 our input considers as predictive attributes the values of the 9 most suitable molecular descriptors and as target attribute the value of FEB obtained from the molecular docking simulation between the receptor and a determined ligand. For experiment 2 our input considers as predictive attributes the same 9 molecular descriptors which are presented in Section 2.3.1.  Table 1 illustrates our data mining inputs.

In our case study we choose to apply the C4.5 [12] classification decision tree algorithm using the J48 implementation on WEKA package [11]. However, the J48 classification algorithm requires a categorical target attribute instead of a continuous one. Since the FEB value is continuous we need to discretize its values. We discretize these values using two different methodologies: by equal frequency (Method 1) and by equal width (Method 2) [8]. Moreover, we split the FEB in 2 (Good and Bad), 3 (Good, Regular, and Bad), and 4 classes (Excellent, Good, Regular, and Bad) for experiments 1 and 2. Thus for each experiment in case study we generated 6 input files: 3 files for each discretization method.

## 3. Results and Discussion

In order to validate the new strategy proposed we performed two case studies: experiment 1 is applied to validate our architecture and experiment 2 is used to generate a set of interesting rules about the performed docking experiments considering as target receptor the protein HIV-1 protease (PDB Code: 1HPV) [23].

From the mining results with the J48 algorithm we evaluate the induced models by typical measures: the rate of correctly classified instances, accuracy (higher values are better), the size of the tree (good values are related to interpretable trees), the root mean-squared error (RMSE), and mean absolute error (MAE) (smaller values are expected). We also considered the *F*-measure (FM), a rate related to the precision, and recall where higher values mean better models. For experiment 2 we also analysed the induced decision trees using the Ordinal Classification (OC) Index metric [31].

### 3.1. Experiment 1: Validating Our Infrastructure

As we mentioned before we induced 6 decision tree models using J48 algorithm with default parameters of execution: discretizing the FEB value in 2, 3, and 4 classes and considering the two discretization methods, by equal frequency (Method 1) and by equal width (Method 2) for each case.  Table 2 summarizes the experiments' results considering the 6 prepared input files.

For discretization in 2 classes, we obtained the same results for Methods 1 and 2 since they induced classes that have about the same number of instances in the two cases. These were the best models with an accuracy of 75% and an interpretable final tree model. Observing the evaluation measures of the 3 classes' inputs, discretization of Method 2, by equal width, obtained better results than discretization by equal frequency. With respect to the 4 classes' inputs, we can see the worst accuracy values for both discretization methods.

**Figure 3:**
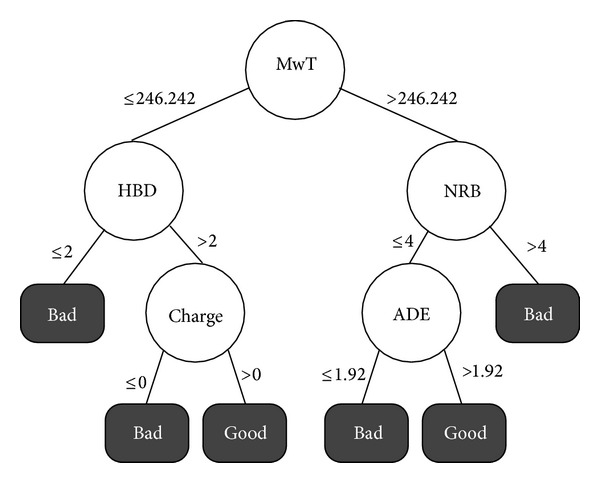
Decision tree induced for the HIV-1 protease with 2 classes considering 9 molecular descriptors.

The induced decision tree model with two classes is depicted in Figure 3. By this figure we can say that there are two rules to determine if a docking experiment is capable of producing good estimated FEB value for the HIV-1 protease.

- The first rule indicates that ligands having the molecular weight lower than 246 mole have chance to be promising if they have over 2 hydrogen bonds donors as well as present a positive charge.
- In the other hand, if we look for bigger ligands, that is, ligands with a molecular weight larger than 246 moles, it is necessary that they have a flexibility with less than 4 numbers of rotatable bounds and an apolar desolvation energy lower than 1.92 kcal/mol.

### 3.2. Experiment 2: Generating Ligand Selection Rules

In this VS experiment we considered a new set of 410 ligands and the same HIV-1 protease as receptor. We also prepared the same 6 input files as detailed on Table 3 and evaluate the induced decision trees with the same metrics of experiment 1.

In order to better analyse the induced decision trees we applied the metric Ordinal Classification (OC) Index [31]. The OC metric is a form to evaluate a multiclass classification for which there is an inherent order between the classes but not a meaningful numeric difference [31]. This is exactly what we have for FEB discretization. This alternative OC measure considers the generated confusion matrix to calculate an error coefficient that should capture how much a result diverges from the ideal prediction and how much the classifier is inconsistent about a relative order of the classes. For instance, if we consider the following conditions: (i) the input file with 3 discretized FEB values (Good, Regular, and Bad); (ii) some instance has the value Good for the target FEB attribute and (iii) the induced decision tree incorrectly predicts the class of this instance. In this case it will be better to classify the instance as Regular rather than as Bad. Also, considering that the induced decision tree incorrectly predicts the class of this instance it will be better to classify it as Regular rather than as Bad. These are the errors that are computed by the OC measure.

**Figure 4:**
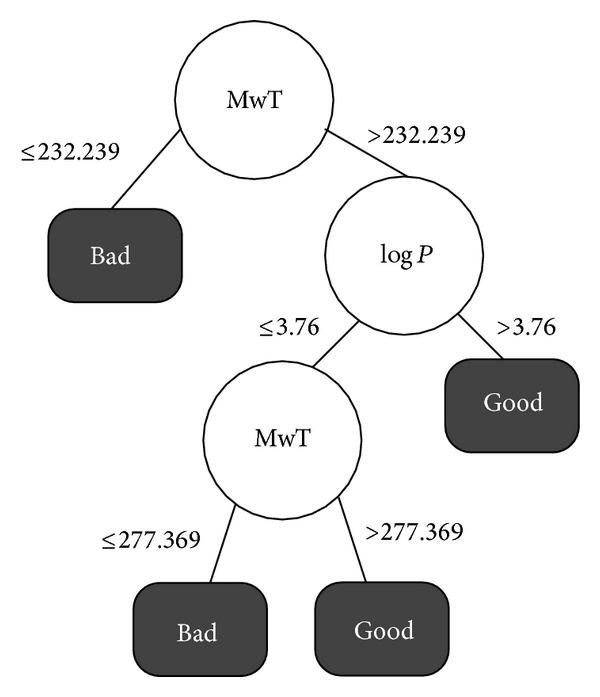
Experiment 2: decision tree induced for the HIV-1 protease with 2 classes, discretizing method by equal width.

Analysing Tables 3 and 4 we can notice that the best results for 2, 3, and 4 classes for both accuracy, *F*-measure, and OC are obtained when considering the discretization Method 2, by equal width. Between these results the best one is for two classes of FEB: Good and Bad as detailed on Figure 4.

**Figure 5:**
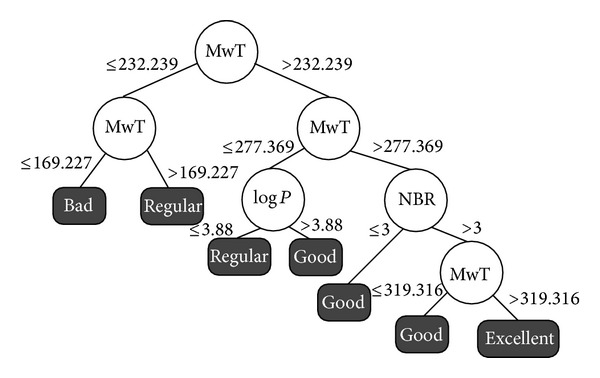
Experiment 2: decision tree induced for the HIV-1 protease with 4 classes, discretizing method by equal width.

Although the best results are obtained with 2 classes, it is also important to analyze the rules related to Excellent FEB values. Thus, we choose to extract rules form the decision tree obtained with the four classes and discretization Method 2 by equal width detailed on Figure 5.

Analysing the induced decision trees of experiment 2 and including the results with 3 classes we can see that the molecular descriptors that are more frequent on these trees are the molecular weight (MwT), log⁡*P*, number of rotatable bonds (NRB), and charge. We can conclude that for this target receptor these molecular characteristics are directly related to Good and Excellent FEB values.

### 3.3. Ligands Selection

The main objective of this work is to propose a methodology to select the most promising ligands for a target receptor. In order to achieve that we use molecular descriptors characteristics obtained from decision trees induced from molecular docking experiments results of a small set of ligands. Following our proposed methodology (Figure 2) the next step is the ligand selection using the rules extracted from the obtained decision trees. We choose to consider the rules obtained from experiment 2 considering 2 and 4 classes and the discretization Method 2.

First, to compare our selection results, we choose the drug-like subset from ZINC database with 15,798,630 compounds in the August of 2013 (https://zinc.docking.org/browse/subsets/). The rules used by ZINC to generate this subset [32] are detailed on Table 5.

We start our ligands selection from the subset detailed on Table 5. We modify in these parameters only the values indicated by the selected rules. In Table 6 we resume the selected rules of Good and Excellent from the decision trees detailed on Figures 4 and 5. We performed the ligands selection using the PubChem [22] interface but choosing only the data from ZINC database [4].

From the results described on Table 6 we notice that the best rule for ligand's selection is MwT between 232.239 and 277.369 mole and log⁡*P* greater than 3.88. This rule selected 33,211 drug-like molecules from ZINC. It corresponds to 0.21% of the total number of ligands in this database, being an effective rule to reduce the number of ligands to be considered on VS experiments for this target receptor.

The other rules for Good class selected from 4.02% (fifth line on Table 6) to 25.34% (second line on Table 6) of the total number of ligands in the drug-like subset database, also being interesting rules to reduce the number of ligands to be used on VS experiments for this target receptor. Considering the unique rule for the class Excellent about 6.61% of the drug-like ligands were selected. Although the number of selected ligands for the rules is still high (from 33,211 to 4,003,380), these initial induced rules effectively reduced set of ligands to be explored in VS experiments validating our proposal methodology.

## 4. Conclusions

Molecular docking simulations can be viewed as one of the most important steps in RDD to accelerate the new drugs discovery process. However, since ligands databases are daily growing, it is in a way impossible to test all the available compounds for a target receptor. In this context it is essential to select the most promising compounds before testing them* in silico*. Thus, in this article, we have presented an approach to mine molecular descriptors data for virtual screening, having a DW schema as support for providing mining inputs. We presented a methodology that makes use of ligands and protein data obtained from public databases, relating the ligands molecular descriptors to virtual screening experiments. These data were properly stored in a DW built for this subject, which is able to produce suitable inputs for the task of mining this kind of data.

To validate our proposal, we performed virtual screening considering as a target receptor the HIV-1 protease and 76 known promising ligands for this protein. Our mining inputs contain molecular descriptors as predictive attributes and the estimated FEB value obtained from docking experiments as the target one. We choose to apply the J48 decision tree algorithm on WEKA [11]. However, this algorithm requires a categorical target attribute instead of a continuous one. Thus we discretized FEB values by equal frequency (Method 1) and by equal width (Method 2). Moreover, we split the FEB in 2 (Good and Bad), 3 (Good, Regular, and Bad), and 4 classes (Excellent, Good, Regular, and Bad).

Experiment 2 was performed to generate a set of interesting rules about the performed docking experiments to be used on ligand selection step. In this VS experiment we considered a new set of 410 ligands and the same receptor preparing the same input data mining files. Analysing the results, we can notice that the best models are for discretization Method 2 and FEB separated in two classes. Besides, we can see that the most frequent molecular descriptors that appear on trees are the MwT, log⁡*P*, NRB, and charge.

Following our proposed methodology, the next step was the ligand selection using the rules extracted from the obtained decision trees. We choose to consider the rules obtained from experiment 2 considering 2 and 4 classes and the discretization Method 2. We choose the drug-like subset from ZINC database with more than 15 million compounds to apply our selection rules using the PubChem [22] interface but choose as source the ZINC database [4]. From the results we notice that the best rule for ligand's selection is selected 0.21% of the total number of ligands in this database, being an effective rule to reduce the number of ligands to be considered on VS experiments for this target receptor.

We intend to further perform VS experiments considering more ligands and refine the generated rules. Besides, we are managing to apply this proposed methodology into the selection of new promising compounds to be* in silico* tested for different protein targets. For instance, we have performed molecular docking simulations considering as target the AcrB protein (PDB Code: 1IWG) present in the plasmatic membrane [33]. Membrane transport proteins are part of the drug efflux and are an important mechanism of bacterial resistance to multiple antibiotics and biocides [34]. Thus it is very important to find promising drug candidates to inhibit this protein. After the molecular docking simulation we will be able to store these data in the proposed DW and we will apply all the methodologies described in this work in order to help to find a set of new promising ligands to be* in silico* analyzed.

## Figures and Tables

**Table 1:** Example of data mining input file format. Column 1 represents the ligand identification (not used on data mining experiments). Columns MwT, log⁡*P*, HBD, HBA, and so forth correspond to the molecular descriptors for each ligand, our predictive attributes. The last column is the target attribute FEB.

Ligand	MwT	log⁡*P*	HBD	HBA	⋯	FEB
1	297.44	4.61	1	2	⋯	−8.50
2	348.47	3.82	2	4	⋯	−7.96
⋯	⋯	⋯	⋯	⋯	⋯	⋯
*N*	200.19	0.54	2	5	⋯	−6.89

**Table 2:** Evaluation metric results of the first set of data mining experiments for validating the proposed architecture. Columns 1 and 2 detail the decision tree experiment. Column 3 corresponds to the accuracy value of the respective decision tree. Column 4 is the size of the tree. Columns 5 and 6 are the RMSE and MAE metrics. Column 7 is the *F*-measure obtained in each induced decision tree.

Classes	Method	Accuracy	Size	RMSE	MAE	FM
2	1	75	11	0.45	0.27	0.75
	2	75	11	0.45	0.27	0.75
3	1	61.84	19	0.47	0.27	0.62
	2	73.32	18	0.44	0.21	0.73
4	1	58.78	17	0.36	0.25	0.59
	2	64.47	19	0.40	0.19	0.65

**Table 3:** Evaluation metric results of the second set of data mining experiments for generating rules about the molecular descriptors. Columns 1 and 2 are the definition of the decision tree experiment characteristics. Columns 3–7 correspond to the resulted metrics for each performed experiment: accuracy, size, RMSE, MAE, and *F*-measure, respectively.

Classes	Method	Accuracy	Size	RMSE	MAE	FM
2	1	84.15	9	0.35	0.22	0.84
	2	84.39	7	0.34	0.20	0.84
3	1	64.88	9	0.39	0.28	0.65
	2	77.81	13	0.33	0.20	0.78
4	1	58.78	17	0.36	0.25	0.59
	2	68.29	13	0.34	0.22	0.67

**Table 4:** Evaluation of the obtained decision trees using the metric Ordinal Classification Index. Columns 1 and 2 describe the performed decision tree experiment and Column 3 describes the value of OC for each experiment where lower values indicate better confusion matrix result.

Classes	Method	OC
2	1	0.26375
	2	0.26051
3	1	0.47436
	2	0.31321
4	1	0.53126
	2	0.37965

**Table 5:** Molecular descriptors rules of the drug-like subset from ZINC database. In Column 1 are the molecular descriptors and in Columns 2 and 3 are the minimum and maximum values for each descriptor.

Descriptor	Minimum	Maximum
Molecular weight (MwT)	150	500
log⁡*P*	−4	5
Number of HBD	0	10
Number of HBA	0	10
Number of rotatable bonds (NRB)	0	8
Apolar desolvation energy (ADE)	−100	40
Polar desolvation energy (PDE)	−400	1
Total polar surface area (TPSA)	0	150
Charge (Ch)	−5	5

**Table 6:** Ligands selection results considering the rules induced by decision trees. Column 1 describes the experiment that induced the decision tree. Column 2 describes the extracted rules. Column 3 shows the respective class of each selected rule and Column 4 describes the total number of selected ligands according to each rule.

Tree	Rules	Class	Selection
2 classes Method 2	MwT > 232.239 and log⁡*P* > 3.76	Good	1,945,022
	MwT > 277.369 and log⁡*P*≤ 3.76	Good	4,003,380

4 classes Method 2	MwT > 232.239, MwT ≤ 277.369, and log⁡*P* > 3.88	Good	33,211
	MwT > 277.369 and NBR ≤ 3	Good	947,028
	MwT > 277.369, MwT ≤ 319.313, and NBR > 3	Good	634,513
	MwT > 319.316 and NBR > 3	Excellent	1,043,884

## Abbreviations

ACC: Accuracy
ADE: Apolar desolvation energy
AIDS: Acquired immunodeficiency syndrome
DW: Data warehouse
FEB: Free energy of binding
FM: *F*-measure
HBA: Number of hydrogen bond acceptors
HBD: Number of hydrogen bond donors
HIV: Human immunodeficiency virus
log⁡*P*: Predicted octanol-water partition coefficient
MAE: Mean absolute error
MwT: Molecular weight
NRB: Number of rotatable bonds
OC: Ordinal Classification Index
PDB: Protein Data Bank
PDE: Polar desolvation energy
RDD: Rational drug design
RMSD: Root mean square deviation
RMSE: Root mean squared error
TPSA: Total polar surface area
VS: Virtual screening.
